# Are PEEK-on-Ceramic Bearings an Option for Total Disc Arthroplasty? An In Vitro Tribology Study

**DOI:** 10.1007/s11999-016-5041-7

**Published:** 2016-09-27

**Authors:** Ryan Siskey, Lauren Ciccarelli, Melissa K. C. Lui, Steven M. Kurtz

**Affiliations:** 1 Exponent, Inc, 3440 Market Street, Suite 600, Philadelphia, PA 19104 USA; 2 Simplify Medical, Sunnyvale, CA USA

## Abstract

**Background:**

Most contemporary total disc replacements (TDRs) use conventional orthopaedic bearing couples such as ultrahigh-molecular-weight polyethylene (polyethylene) and cobalt-chromium (CoCr). Cervical total disc replacements incorporating polyetheretherketone (PEEK) bearings (specifically PEEK-on-PEEK bearings) have been previously investigated, but little is known about PEEK-on-ceramic bearings for TDR.

**Questions/purposes:**

(1) What is the tribologic behavior of a PEEK-on-ceramic bearing for cervical TDR under idealized, clean wear test conditions? (2) How does the PEEK-on-ceramic design perform under impingement conditions? (3) How is the PEEK-on-ceramic bearing affected by abrasive wear? (4) Is the particle morphology from PEEK-on-ceramic bearings for TDRs affected by adverse wear scenarios?

**Methods:**

PEEK-on-ceramic cervical TDR bearings were subjected to a 10 million cycle ideal wear test based on ASTM F2423 and ISO 181912-1 using a six-station spine wear simulator (MTS, Eden Prairie, MN, USA) with 5 g/L bovine serum concentration at 23° ± 2° C (ambient temperature). Validated 1 million cycle impingement and 5 million cycle abrasive tests were conducted on the PEEK-on-ceramic bearings based, in part, on retrieval analysis of a comparable bearing design as well as finite element analyses. The ceramic-on-PEEK couple was characterized for damage modes, mass and volume loss, and penetration and the lubricant was subjected to particle analysis. The resulting mass wear rate, volumetric wear rate, based on material density, and particle analysis were compared with clinically available cervical disc bearing couples.

**Results:**

The three modes of wear (idealized, impingement, and abrasive) resulted in mean mass wear rates of 0.9 ± 0.2 mg/MC, 1.9 ± 0.5 mg/MC, and 2.8 ± 0.6 mg/MC, respectively. The mass wear rates were converted to volumetric wear rates using density and found to be 0.7 ± 0.1 mm^3^/MC, 1.5 ± 0.4 mm^3^/MC, and 2.1 ± 0.5 mm^3^/MC, respectively. During each test, the PEEK endplates were the primary sources of wear and demonstrated an abrasive wear mechanism. Under idealized and impingement conditions, the ceramic core also demonstrated slight polishing of the articulating surface but the change in mass was unmeasurable. During abrasive testing, the titanium transfer on the core was shown to polish over 5 MC of testing. In all cases and consistent with previous studies of other PEEK bearing couples, the particle size was primarily < 2 µm and morphology was smooth and spheroidal.

**Conclusions:**

Overall, the idealized PEEK-on-ceramic wear rate (0.7 ± 0.1 mm^3^/MC) appears comparable to the published wear rates for other polymer-on-hard bearing couples (0.3–6.7 mm^3^/MC) and within the range of 0.2 to 1.9 mm^3^/MC reported for PEEK-on-PEEK cervical disc designs. The particles, based on size and morphology, also suggest the wear mechanism is comparable between the PEEK-on-ceramic couple and other polymer-on-ceramic orthopaedic couples.

**Clinical Relevance:**

The PEEK-on-ceramic bearing considered in this study is a novel bearing couple for use in total disc arthroplasty devices and will require clinical evaluation to fully assess the bearing couple and total disc design. However, the wear rates under idealized and adverse conditions, and particle size and morphology, suggest that PEEK-on-ceramic bearings may be a reasonable alternative to polyethylene-on-CoCr and metal-on-metal bearings currently used in cervical TDRs.

## Introduction

Fixed-bearing and mobile-bearing cervical total disc replacements (TDRs) that incorporate traditional orthopaedic bearing materials such as ultrahigh-molecular-weight polyethylene (hereafter, polyethylene) and cobalt-chromium (CoCr) alloys have been used for several decades to treat patient pain associated with degenerative disc disease [34]. Polyethylene, CoCr, and stainless steel bearings for cervical TDR were approved for clinical use based on idealized, clean wear rates obtained from standardized testing [2, 3, 5, 11, 20–25, 27, 29, 34–36, 43]. Current industry standards for evaluating wear of TDRs are limited to idealized wear conditions, encompassing the intended bearing surfaces under anatomically justified ROMs. However, little is known about the tribologic behavior of cervical TDRs under adverse wear conditions such as impingement or third-body wear [36].

Recently, polyetheretherketone (PEEK) has been considered as an alternative bearing for all-polymer cervical TDRs [7, 9–11, 27, 31, 32, 51]. PEEK bearings are attractive candidates for TDR because they avoid the use of metallic components and do not interfere with MRI. However, under elevated contact stresses in vitro, a delamination wear mechanism has been observed in PEEK-on-PEEK TDRs [27].

PEEK-on-ceramic bearings represent another alternative for cervical TDRs. Although ceramic biomaterials are well accepted in large joint replacements [13, 15, 19, 38, 40, 45, 46, 49, 50], fracture risk is a concern [1, 44]. It is also unclear if PEEK-on-ceramic bearings will demonstrate acceptably low wear under idealized or aggressive tribologic conditions. Orthopaedic bearings may be exposed to adverse wear conditions that differ from the idealized conditions currently prescribed by industry standards. Cervical TDR polymer-on-ceramic bearings are no exception. THAs are assessed preclinically under conditions meant to simulate impingement based on retrieval studies that have shown ceramic femoral heads with evidence of metal transfer consistent with subluxation and/or impingement [6, 14, 17, 18, 30, 39, 41, 42]. Similarly, for TDRs, many designs have been shown to demonstrate impingement [28, 35, 36]. Therefore, assessing the effect of both impingement and metal transfer on a candidate polymer-on-ceramic bearing couple is essential. Furthermore, the particles generated under these adverse conditions may be different than the idealized conditions and therefore warrant investigation.

Consequently, we addressed the following research questions in a series of experiments: (1) What is the tribologic behavior of a PEEK-on-ceramic bearing for cervical TDR under idealized, clean wear test conditions? (2) How does the PEEK-on-ceramic design perform under impingement conditions? (3) How is the PEEK-on-ceramic bearing affected by abrasive wear? (4) Is the particle morphology from PEEK-on-ceramic bearings for TDRs affected by adverse wear scenarios?

## Materials and Methods

**Fig. 1A–E:**
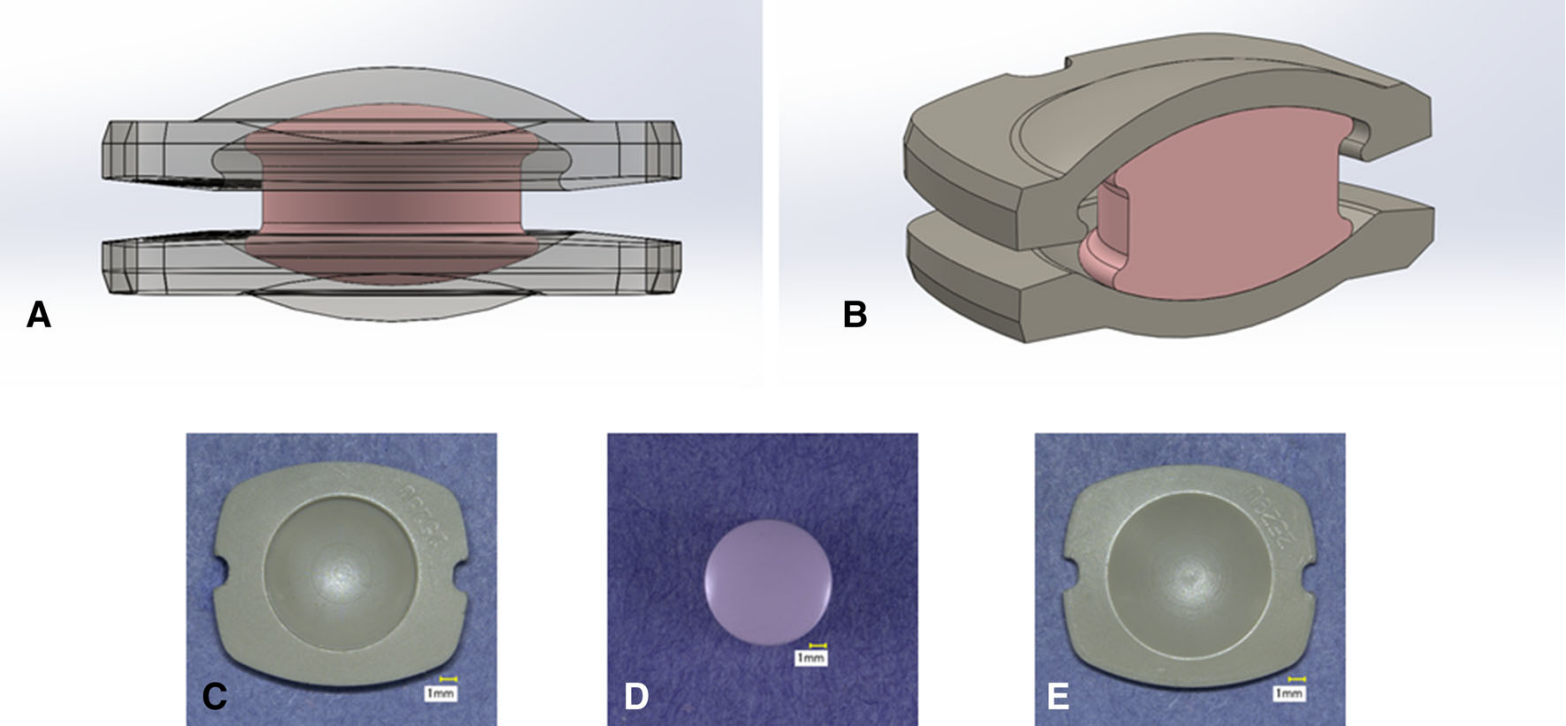
(**A**–**B**) Representative assembly schematics show the wear coupons used for testing. (**C**–**E**) Digital images showing the individual implant components: (**C**) superior PEEK endplate; (**D**) ceramic core; (**E**) inferior PEEK endplate.

Test coupons were created for this study to be representative of an investigational mobile-bearing design (Simplify Medical, Sunnyvale, CA, USA). The geometry of this design is based on the KineFlex^®^|C Cervical TDR (SpinalMotion, Mountain View, CA, USA). The test coupons in the present study were comprised of two PEEK endplates (PEEK^®^ Optima Natural; Invibio, West Conshohocken, PA, USA) separated by a mobile zirconia-toughened alumina ceramic core (CeraSurf^®^-p; CoorsTek^®^ Medical, Grand Junction, CO, USA) (Fig. 1). The titanium plasma spray coating and other surface features that are normally present on the bone-contacting surfaces of the endplates were omitted per ASTM F2423 for ease of fixturing and to preclude third-body wear from compromising the evaluation of the articulating surfaces. The endplate footprints were all 14 × 16 mm with the exception of the impingement wear samples. Two sizes, small (12 × 15 mm) and large (16 × 18 mm), were evaluated under impingement conditions. All samples were subjected to 25 kGy terminal sterilization before testing.

Wear testing was conducted using a six-station spine wear simulator (MTS, Eden Prairie, MN, USA). Wear testing parameters for the idealized, impingement, and abrasive wear testing were established using ASTM F2423 and ISO 18192-1 as guides (Table 1) [5, 29]. A preliminary study using idealized conditions was performed to assess the effect of bovine serum concentration and solution temperature. A 1.0 million cycle (MC) test was used to assess the wear rate of the PEEK-on-ceramic bearing using the standard 20 g/L bovine serum concentration at 37° ± 2° C and a reduced concentration of 5 g/L at 23° ± 2° C (ambient temperature). Three bearings were evaluated under each lubrication condition, stopping the testing at 0.25 MC intervals to clean and inspect the bearing surfaces for wear mechanisms and to determine the mass loss of each component. The volumetric loss was calculated by dividing the mass loss of the entire device by the density of PEEK (1.3 mg/mm^3^). This assumes that the majority of the mass loss is coming from the PEEK endplates and not the ceramic cores. No difference was found in wear rate between the two lubrication conditions: 2.2 ± 0.3 mg/MC (37° C, 20 g/L) versus 2.4 ± 0.4 mg/MC (23° C, 5 g/L; t-test p = 0.76). Under both lubrication conditions, the endplates demonstrated microabrasive polishing and the core demonstrated no measurable change in mass. Therefore, the idealized wear test, using the reduced concentration/ambient temperature condition, was carried out on six samples to 10 MC, stopping at 0.25 MC intervals up to 1.0 MC and 0.5 MC intervals thereafter.

**Table 1:** The table summarizes the wear testing parameters by wear test scenario

Motions and loading profiles	Wear test parameters		
	Idealized*	Impingement*	Abrasive*
Flexion/extension (degrees)	± 7.5	17–18^†^	± 7.5
Lateral bending (degrees)^‡^	± 6	N/A	± 6
Axial rotation (degrees)^‡^	± 6^§^	± 6^§^	± 6^§^
Axial load (N)|	50–150	150	50–150

**Fig. 2A–D:**
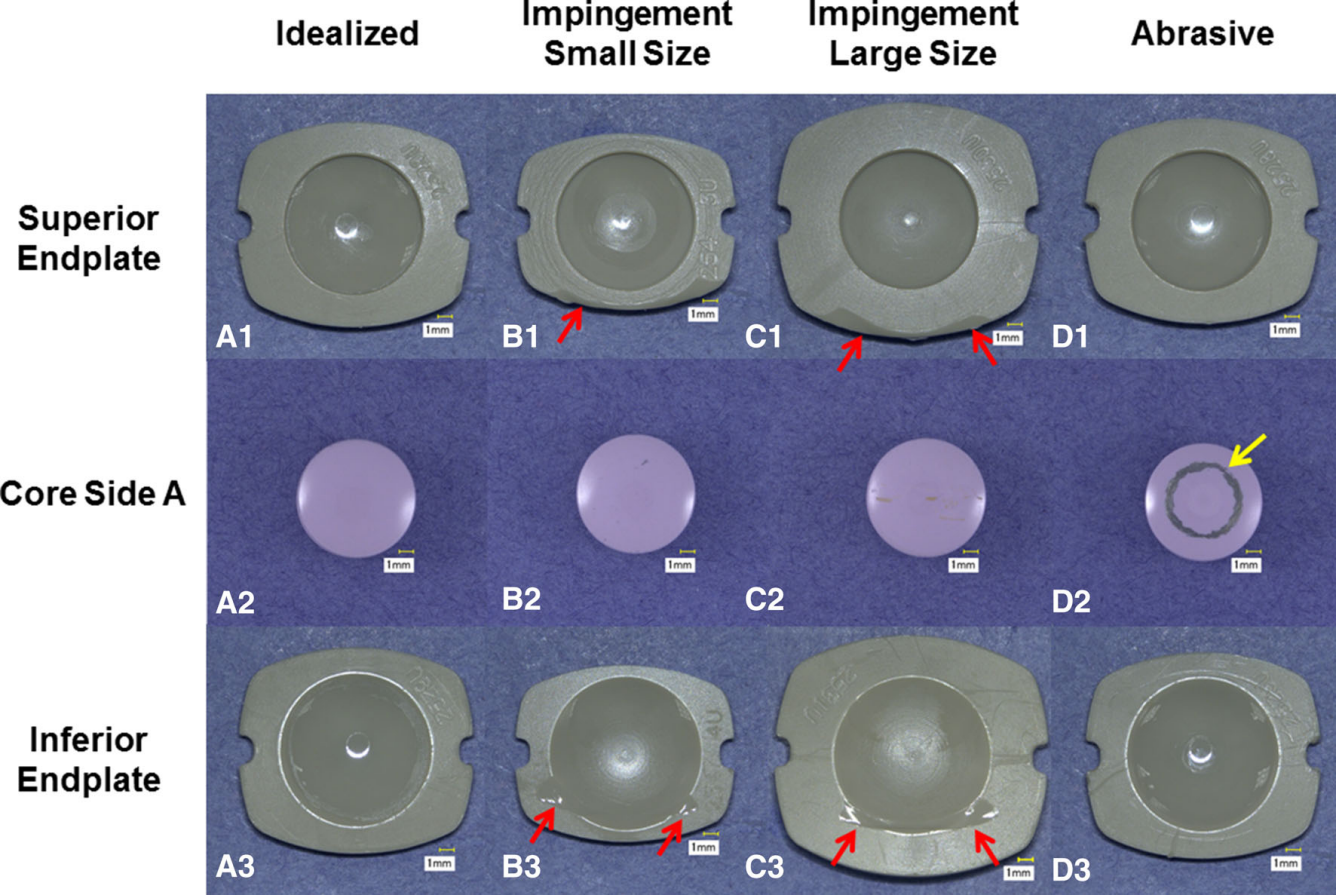
(**A1**–**A3**) Representative superior endplate, core, and inferior endplate after idealized wear testing are shown. (**B1**–**B3**) Representative superior endplate, core, and inferior endplate after impingement wear testing for the small size components. (**C1**–**C3**) Representative superior endplate, core, and inferior endplate after impingement wear testing for the large size components. The red arrows in images **B1**–**B3** and **C3** indicate the region of impingement. (**D1**–**D3**) Representative superior endplate, core, and inferior endplate after abrasive wear testing. The yellow arrow in **C2** indicates the titanium transfer applied to the core for abrasive testing.

* For each test, six samples were evaluated; for impingement testing, the six samples consisted of three small and three large samples; ^†^this includes a 10° extension bias of the fixtures; ^‡^lateral bending was shifted 90° relative to flexion/extension and shifted 180° relative to axial rotation per Figure 2 found in ISO 18192-1; flexion/extension and axial load were in phase; ^§^the magnitude of axial rotation as specified in ASTM F2423 was used in this test because it was more severe than the ± 4° magnitude specified in ISO 18192-1; |the magnitude of axial load as specified in ISO 18192-1 was used in this test because the peak load is more severe than the 100-N magnitude specified in ASTM F2423.

Impingement wear testing was conducted on three samples of two different size devices (six total) representing the smallest and largest available footplate geometries. Using a validated cervical spine finite element model, radiographs from retrieval studies of the KineFlex^®^|C Disc [16], and manufacturer-provided models of the Simplify Disc, the device was evaluated to determine the point at which impingement occurred in extension. Because no published standard exists for impingement testing, a validation experiment, using sample KineFlex^®^|C Discs, was conducted using the specified impingement motions and loading that were used in the experimental wear testing (Table 1). The resulting wear patterns on the KineFlex^®^|C Disc were found to mimic the wear patterns from in vivo retrieved KineFlex^®^|C devices, supporting the impingement testing protocol employed in this study. The three-dimensional models of the Simplify Disc were then used to assess the different footprint geometries and to determine the approximate angles of impingement. The smallest and largest endplates were selected as the worst-case contact stress and contact area impingement scenarios, respectively. After the impingement conditions were determined, a 1.0 MC test using the reduced concentration/ambient temperature condition was conducted with interval analysis at 0.05, 0.1, 0.25, 0.5, 0.75, and 1.0 MC.

Abrasive wear testing was conducted on six samples using the same parameters and sample geometry as described for the idealized testing. The test was conducted to 5 MC, stopping at 0.25 MC intervals up to 1.0 MC and 0.5 MC intervals thereafter. A titanium transfer scar, rather than slurry with particulate or scratched counterface [8, 26, 49], was used to create the abrasive tribologic test conditions. The titanium transfer scar was deposited on six cores before the test by articulating a titanium Grade 4 rod against both articulating faces of the ceramic cores. The motion of the rod was controlled axially using a servohydraulic load frame (858 MiniBionix II; MTS, Eden Prairie, MN, USA), while the core was rotated, to create circular deposits around the axis of symmetry of cores. The 10-point height (Rz) of titanium transfer on cores was monitored at each interval analysis through white light interferometry to ensure titanium transfer was still present.

At each interval, of each test, the samples were inspected for wear and damage modes and measured for mass loss. The PEEK endplates were imaged using a μCT 80 (Scanco Medical AG, Brüttisellen, Switzerland) at a maximum voxel resolution of 20 μm before the start and at the end of each test. Custom Matlab code (MathWorks, Natick, MA, USA) was developed to calculate the dimensional changes of the PEEK bearing surface and to estimate penetration [47]. This analysis resulted in a penetration map, which was used to visualize the combination of wear and deformation across the entire surface of the endplates.

For each mode of testing (ideal, impingement, abrasive), the mass loss by cycle count was tested to ensure it was normally distributed. The corresponding volume loss was determined using the density of the PEEK material (1.3 mg/mm^3^). Once normality was confirmed, the mass loss was analyzed using a regression analysis to assess linearity. The mean and SD mass wear rate were calculated for each mode of testing and compared using a Student’s t-test. For the regression analysis and Student’s t-test, a p value ≤ 0.05 was used to assess statistical significance. JMP 12.0.1 was used to conduct all statistical analyses (SAS, Cary, NC, USA).

Wear particle analysis was conducted using three bovine serum samples obtained at the final interval of each test scenario. The bovine serum samples were selected based on the mass loss of the devices; the serum from the stations demonstrating the average wear rate and two highest wear rates were used. Particle size, shape, and morphology were determined according to ASTM F1877 [4]. Enzymatic digestions were performed. Each serum sample was shaken by hand for 60 seconds, after which 10 mL of serum was removed from each sample and added to 100 mL of a 1% solution of enzyme in deionized water. The samples were digested at 37° C for a minimum of 3 days with periods of intermittent ultrasonication. After digestion, a 40 mL aliquot of the digested fluid was removed; each aliquot was added to 50 mL deionized water. The samples were passed through a 1 µm polycarbonate membrane (Whatman, Kent, UK). The filtrate was reserved and subsequently passed through a 100 nm polycarbonate membrane (Whatman, Kent, UK). Filters were gold/palladium sputtercoated (30 mA for 30 seconds) using a sputtercoater (208HR; Cressington, Watford, UK). Scanning electron microscopy was performed using an analytical scanning electron microscope (SEM) (Quanta 600 ESEM; FEI, Hillsboro, OR, USA). Coated samples were inserted into the SEM for imaging under high-vacuum mode at 5 kV. All 0.1- and 1.0-µm filters were imaged at 10,000× and 1000× magnification, respectively. For each filter, five fields of view were captured for image analysis. Each SEM micrograph was thresholded and then processed using a custom script in NIH ImageJ (National Institutes of Health, Bethesda, MD, USA). The particles were counted and, based on the scale bar in each SEM micrograph, a particle area was determined. Energy-dispersive X-ray spectroscopy (EDS) was used to determine representative particle elemental composition. The resulting particle equivalent circular diameter was plotted as a frequency distribution for each wear mode analyzed.

## Results

**Fig. 3:**
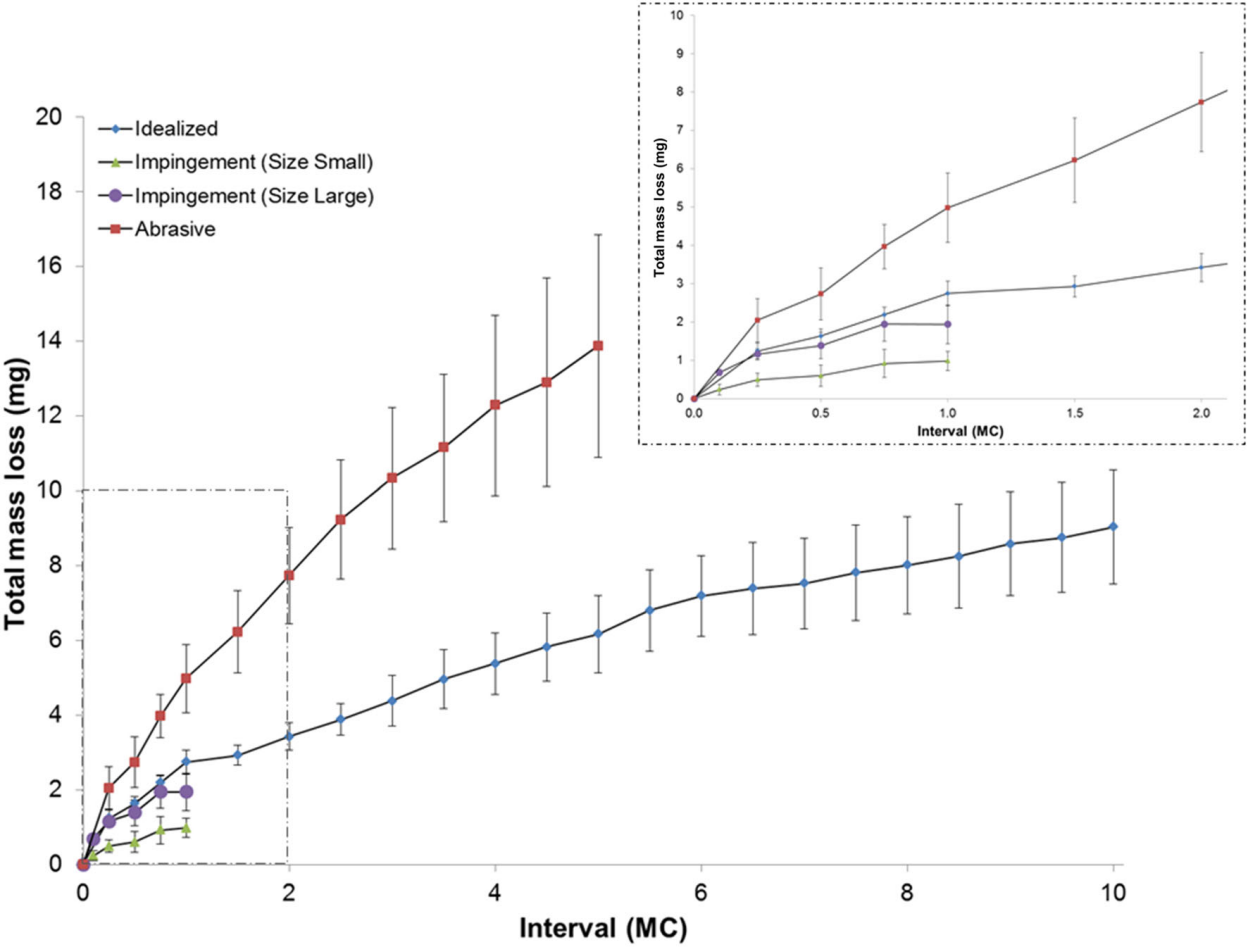
Graph depicts the total mass loss versus number of cycles for each wear mode evaluated. The top graph shows all intervals and the inset (dashed box) shows only the total mass loss versus number of cycles through 2.0 MC. The data presented at each interval mean ± 1 SD of n = 6 devices for ideal and abrasive conditions and n = 3 for each impingement test.

**Fig. 4A–D:**
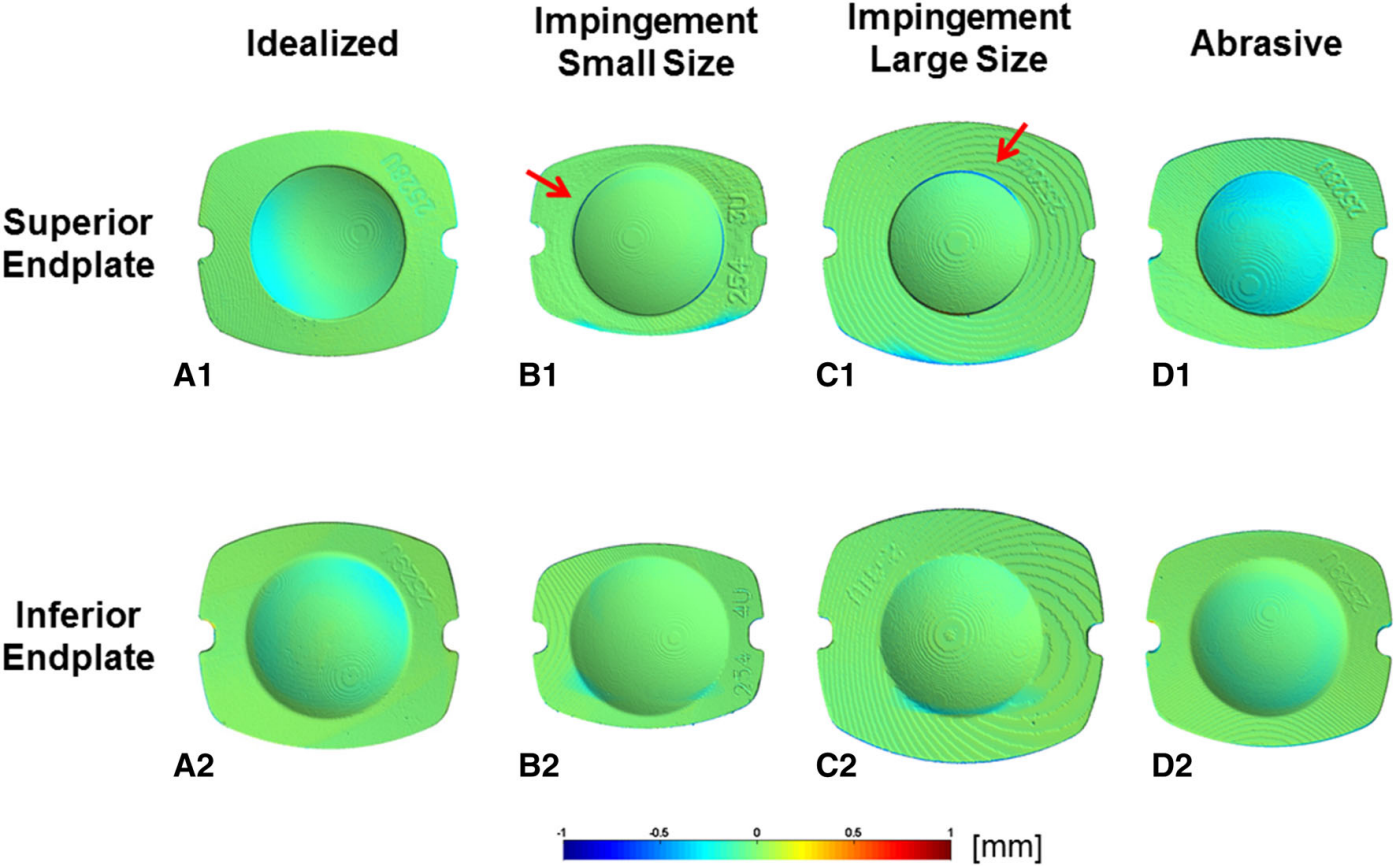
The representative penetration maps (**A1**–**A2**) show the superior and inferior endplates after idealized wear testing. The representative penetration maps (**B1**–**B2**) of the superior and inferior endplates after impingement wear testing for the small size components. The representative penetration maps (**C1**–**C2**) of the superior and inferior endplates after impingement wear testing for the large size components. The computational artifact present at the retention ring (dark blue ring indicated by the red arrows in **B1** and **C1**) is not representative of actual penetration. The representative penetration maps (**D1**–**D2**) show the superior and inferior endplates after abrasive wear testing.

The wear performance of a PEEK-on-ceramic TDR bearing under idealized, standard cervical disc conditions resulted in a microabrasive wear mode of the PEEK endplates and subtle abrasive polishing of the ceramic core (Fig. 2). The endplates demonstrated no evidence of fracture or risk of endplate penetration resulting from wear under these conditions. The ideal mass loss across the six samples was confirmed to be normally distributed. The mean wear rate for 10 MC of testing was found to be 0.9 ± 0.2 mg/MC (mean volumetric wear rate of 0.7 ± 0.1 mm^3^/MC for 10 MC of testing), was linear (R^2^ = 0.86, p < 0.0001), and no samples demonstrated runaway wear (Fig. 3). The PEEK endplates demonstrated measurable mass loss, whereas the mass of the ceramic cores remained constant through 10 MC. The penetration maps of the superior and inferior endplates indicated that the maximum penetration occurred at the articulating surface of the inferior endplate. The average penetration for this region was 0.08 ± 0.03 mm after 10 MC (Fig. 4).

Under impingement wear conditions, both the inferior and superior PEEK endplates, of both size devices, demonstrated impingement wear patches in regions consistent with the contact patches predicted by finite element analysis and solid modeling. These regions, located on the posterior rims of the endplates, were burnished and consistent with a microabrasive mechanism between the PEEK surfaces. Penetration maps confirmed the penetration was generally most apparent in the smaller size and less in the larger size configuration. The maximum penetration at the area of impingement for 1.0 MC ranged from 0.16 mm to 0.33 mm across both sizes of endplates and occurred at the rim away from the intended articulating surface (Figs. 2, 4). Despite this location, the endplates demonstrated no evidence of fracture. The mass loss from the six samples was confirmed to be normally distributed. The mean mass wear rate for 1 MC of impingement testing was linear (Size 1: R^2^ = 0.72, p < 0.0001; Size 3: R^2^ = 0.76, p < 0.0001) and found to be 1.0 ± 0.2 mg/MC and 1.9 ± 0.5 mg/MC for the small and large designs, respectively (Fig. 3). The wear rate for the larger size under impingement conditions was higher than the idealized conditions (0.98; confidence interval [CI], 0.23–1.73; p = 0.014) and comparable for the smaller size (0.13; CI, −0.62 to 0.87; p = 0.724). The volumetric wear rate for the small and large designs was 0.8 ± 0.2 mm^3^/MC and 1.5 ± 0.4 mm^3^/MC, respectively.

Under abrasive conditions, the PEEK endplates demonstrated polishing of the superior articulating surface (Fig. 2). The titanium transfer was confirmed to be present throughout the duration of the 5 MC tests but did demonstrate a reduction in height (Fig. 2). The penetration maps of the superior and inferior endplates indicated that the maximum penetration occurred at the articulating surface of the superior endplate. The mean penetration for this region was 0.13 ± 0.06 mm for 5 MC (Fig. 4). Given the initial thickness of the device is approximately 1 mm, it did not appear the PEEK endplates were readily susceptible to complete wear through under abrasive conditions. The mass loss for the six samples was normally distributed. The mean mass wear rate for 5 MC of abrasive testing was found to be 2.8 ± 0.6 mg/MC, was linear (R^2^ = 0.86, p < 0.0001), and, like the idealized test, no samples demonstrated runaway wear (Fig. 3). The volumetric wear rate for 5 MC of abrasive testing was 2.1 ± 0.5 mm^3^/MC. The abrasive conditions therefore produced the highest wear rate for the PEEK-on-ceramic bearing that was higher than both the idealized and impingement wear rates (abrasive versus ideal: 1.82, CI, 1.21–2.42, p < 0.001; abrasive versus impingement, smaller size: 1.69, CI, 0.94-2.44, p = 0.0003; abrasive versus impingement, larger size: 0.83, CI, 0.087–1.58, p = 0.0313).

**Fig. 5A–C:**
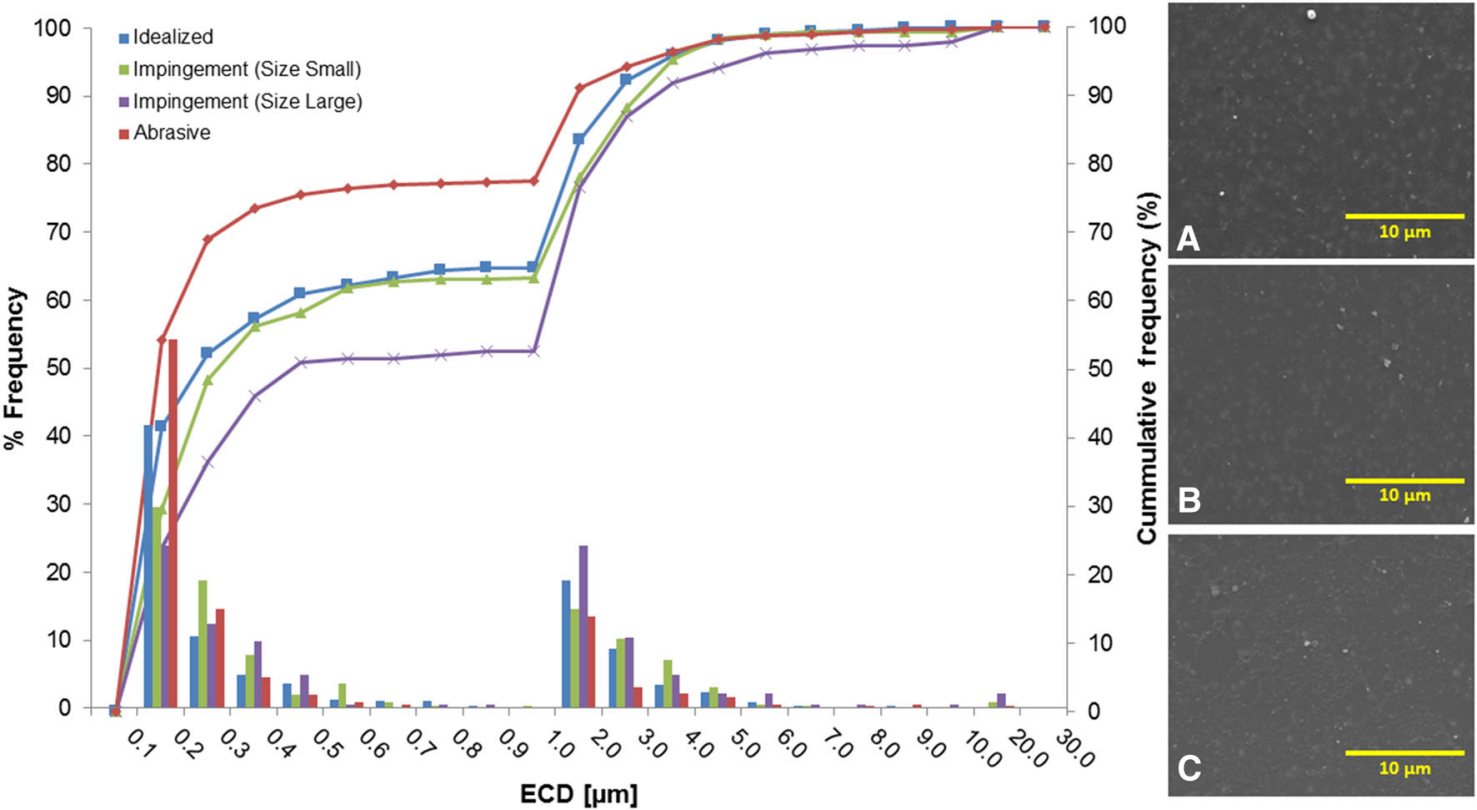
Graph depicts the equivalent circular diameter (ECD) frequency distribution for maximum wear stations from ideal, impingement, and abrasive wear testing. Representative SEM micrographs of particles taken at × 10,000 are shown from the idealized (**A**), impingement (**B**), and abrasive (**C**) wear testing fluid samples.

Particles generated during idealized conditions were similar in size, shape, and morphology to those generated during impingement and abrasive conditions. The size distributions for all three tests were compared and found to have mean equivalent circular diameter values ranging from 0.10 to 16.69 µm (Fig. 5). During the last interval of testing for all modes, the size distributions were bimodal with the two main subgroups of particles found in the 0.1 to 0.2 µm and 1.0 to 2.0 µm size ranges (Fig. 5). Overall, 97% of the particles were less than 5.0 µm in diameter. For the idealized wear test, 65% of the particles produced were less than 1.0 µm and were 0.22 ± 0.15 µm in size. The EDS spectra for the particles for all tests indicated the particles were primarily polymeric with a small number of ceramic particles also present. Using the nomenclature for describing particle morphology according to ASTM F1877, the larger particulate tended to be smooth flakes and globular particles along with a few fibrils. The submicron particulate was primarily smooth with spheroidal granules.

## Discussion

Replacing the diseased cervical disc with an arthroplasty device provides surgeons with an alternative to fusion for some patients. Although many arthroplasty devices are comprised of CoCr or titanium articulating against ultrahigh-molecular-weight polyethylene, other less traditional biomaterials for cervical TDRs may improve the clinical performance of these devices. A PEEK-on-ceramic bearing is an example of a possible bearing combination, but little is known about the wear performance under cervical disc conditions. The results of this study suggest that the PEEK-on-ceramic bearing combination is sufficiently robust and predictable for cervical TDR applications to warrant further investigation in a clinical study. The PEEK-on-ceramic bearing couple demonstrated no evidence of runaway wear, endplate perforation, or component fracture in any of the idealized or aggressive wear simulations.

This study has several limitations. The displacements and loads that were used for idealized and adverse testing were based on testing standards, and therefore the results of wear testing may not be indicative of the full range of tribologic conditions that may be encountered in vivo. Although the wear rate for each set of conditions appears repeatable within each test, the overall sample sizes and limitation of six wear stations make adding statistical power to these studies extremely resource-intensive and impractical. The test coupons used for testing were representative of the final bearing geometry intended for cervical disc replacement; however, the coupons did not have the final backside geometry or titanium plasma spray so the performance of that interface could not be assessed. The impingement and abrasive testing conditions are adverse scenarios that were based on reported methods and observations in the literature [28, 36, 37] and were derived from experience with retrievals and wear testing from other devices. This study did not explore other potential adverse scenarios that could potentially lead to different wear modes or bearing couple performance.

When compared with other cervical disc bearing couples, the idealized PEEK-on-ceramic wear rate (0.7 ± 0.1 mm^3^/MC) appears comparable to the published wear rates for other polymer-on-hard bearing couples (0.3–6.7 mm^3^/MC) and within the range of 0.2 to 1.9 mm^3^/MC reported for PEEK-on-PEEK cervical disc designs (Table 2). The bearing couple demonstrated no evidence of delamination wear as has been shown for some cervical disc PEEK-on-PEEK bearing couples [27].

**Table 2:** Summary of available wear testing data for cervical disc replacements under idealized (Mode I) conditions

Reference	Design	Bearing couple	Standard referenced	Relevant test inputs	Wear rate	Particle size
SSED for PMA Number: P060023 (Bryan^®^ Cervical Disc) [21]	Bryan^®^ (Medtronic Sofamor Danek, Memphis, TN, USA)	Polycarbonate urethane on titanium	N/A	FE: ± 4.9° LB: N/A AR: ± 3.8° AL: 130 N and 300 N F: 2, 4, and 6 Hz	As published: 0.4–1.4 mg/ MC Converted (density = 1.2 mg/mm^3^): 0.3–1.2 mm^3^/MC	Greater than 90% of particles were less than 1 µm
SSED for PMA Number: P070001 (ProDisc™-C Total Disc Replacement) [20]	ProDisc™-C (Synthes Spine, West Chester, PA, USA)	UHMWPE on CoCr	N/A	FE: ± 7.5° LB: ± 6.0° AR: ± 4.0° AL: 150 N F: 1.0 Hz	As published: 2.59 ± 0.36 mg/MC Converted (density = 0.94 mg/mm^3^): 2.8 ± 0.36 mm^3^/MC	Average size 0.17–0.35 µm
Nectow et al., 2008 [43]	ProDisc™-C (Synthes Spine)	UHMWPE on CoCr	ISO 18192-1	FE: ± 7.5° LB: ± 6.0° AR: ± 4.0° AL: 50-150 N F: 1.0 Hz	As published: 1.99 ± 0.15 mg/MC Converted (density = 0.94 mg/mm^3^): 2.12 ± 0.15 mm^3^/MC	Greater than 90% of particles were less than 1 µm
Bushelow et al., 2008 [12]	ProDisc™-C (Synthes Spine)	UHMWPE on CoCr	N/A	FE: ± 5° LB: ± 5.0° AR: ± 3.5° AL: 15-150 N F: 1.0 Hz	As published: 0.88 ± 0.07 mg/MC Converted (density = 0.94 mg/mm^3^): 0.94 ± 0.07 mm^3^/MC	Greater than 90% of particles were less than 1 µm; average diameter between 0.22 and 0.37 µm
			N/A	FE: ± 10° LB: ± 5.0° AR: ± 4.0° AL:150 N F: 1.0 Hz	As published: 2.59 ± 0.36 mg/MC Converted (density = 0.94 mg/mm^3^): 2.75 ± 0.38 mm^3^/MC	
			ISO 18192-1	FE: ± 7.5° LB: ± 4.0° AR: ± 6.0° AL: 15-150 N F: 1.0 Hz	As published: 1.82 ± 0.11 mg/MC Converted (density = 0.94 mg/ mm^3^): 1.94 ± 0.12 mm^3^/MC	
SSED for PMA Number: P110002 (Mobi-C^®^ Cervical Disc Prosthesis) [24]	Mobi-C^®^ (LDR Spine, Austin, TX, USA)	UHMWPE on CoCr	ISO 18192-1	FE: ± 7.5° LB: ± 6.0° AR: ± 4.0° AL: 50-150 N F: 1.0 Hz	As published: 1.55 ± 0.08 mg/MC Converted (density = 0.94 mg/mm^3^): 1.64 ± 0.09 mm^3^/MC	Average size 0.77 µm
SSED for PMA Number: P100003 (SECURE^®^-C Cervical Artificial Disc) [23]	SECURE^®^-C (Globus Medical, Audubon, PA, USA)	UHMWPE on CoCr	N/A	FE: ± 7.0° LB: ± 7.0° AR: ± 1.5° (Test I) and ± 6.0° (Test II) AL: 150 N F: 2.0 Hz	As published: Test I: 2.57 ± 1.21 mg/MC Test II: 0.89 ± 0.3 mg/MC Converted (density = 0.94 mg/mm^3^): Test I: 2.73 ± 1.29 mm^3^/MC Test I: 0.95 ± 0.32 mm^3^/MC	N/A
SSED for PMA Number: P100012 (PCM^®^ Cervical Disc) [22]	PCM^®^ (NuVasive, San Diego, CA, USA)	UHMWPE on CoCr	ISO 18192-1	FE: ± 7.5° LB: ± 6.0° AR: ± 4.0° AL: 50-150 N F: 1.0 Hz	As published: 6.33 mg/MC Converted (density = 0.94 mg/mm^3^): 6.73 mm^3^/MC	Average size: 0.43 ± 0.04 µm and 0.53 ± 0.15 µm for two samples, respectively
Grupp et al., 2015 [28]	activ^®^ C (Aesculap AG, Tuttlingen, Germany)	UHMWPE on CoCr	ISO 18192-1	FE: ± 7.5° LB: ± 6.0° AR: ± 4.0° AL: 50-150 N F: 1.0 Hz	As published: 1.0 ± 0.1 mg/MC Converted (1.29 mg/mm^3^): 0.78 ± 0.08 mm^3^/MC	99% of particles were less than 1 µm
		PEEK on PEEK			As published: 1.4 ± 0.4 mg/MC Converted (1.29 mg/mm^3^): 1.1 ± 0.3 mm^3^/MC	99% of particles were less than 1 µm
		CFR-PEEK on CFR-PEEK			As published: 0.02 ± 0.02 mg/MC Converted (1.29 mg/mm^3^): 0.02 ± 0.02 mm^3^/MC	99% of particles were less than 1 µm
		PEK on PEK			As published: 0.8 ± 0.1 mg/MC Converted (1.29 mg/mm^3^): 0.6 ± 0.1 mm^3^/MC	90% of particles were less than 1 µm
Brown et al., 2010 [11]	NuNec (Pioneer Surgical, Marquette, MI, USA)	PEEK on PEEK	ASTM F2423	FE: ± 7.5° LB: ± 6.0° AR: ± 4.0° AL: 50-150 N F: 2.0 Hz	As published: 0.26 ± 0.01 mg/MC Converted (1.29 mg/mm^3^): 0.20 ± 0.01 mm^3^/MC	N/A
			ISO 18192-1	FE: ± 7.5° LB: ± 6.0° AR: ± 6.0° AL: 100 N F: 2.0 Hz	As published: 0.32 ± 0.02 mg/MC Converted (1.29 mg/mm^3^): 0.25 ± 0.02 mm^3^/MC	N/A
Xin et al., 2013 [51]	NuNec (Pioneer Surgical)	PEEK on PEEK	ISO 18192-1	FE: ± 7.5° LB: ± 6.0° AR: ± 6.0° AL: 50-150 N F: 1.0 Hz	As published: 2.5 ± 0.1 mg/MC As published: 1.9 ± 0.1 mm^3^/MC	N/A
Design History File Report for the Kineflex|C	Kineflex|C (SpinalMotion, Mountain View, CA)	CoCr on CoCr	ISO 18192-1	FE: ± 7.5° LB: ± 6.0° AR: ± 6.0° AL: 100 N F: 5.0 Hz	As published: 3.2 mg/ MC Converted (Density = 8.5 mg/mm^3^): 0.4 mm^3^/MC	Average size: 0.4–0.55 µm
Kurtz et al., 2012 [35]	Prestige ST (Medtronic Sofamor Danek)	Stainless steel on stainless steel	ASTM F2423	FE: ± 9.7° (Phase II) LB: ± 4.7° (Phase I) AR: ± 3.8° (Phase I) AL: 49 N (Phase I)/149 N (Phase II) F: 1.0 Hz	As published: Phase I: 0.72-0.76 mm^3^/MC Phase II: 0.004-0.059 mm^3^/MC	Average size: 0.3–0.6 µm
SSED for PMA Number: P090029 (Prestige^®^ LP Cervical Disc) [25]	Prestige^®^ LP (Medtronic Sofamor Danek)	Titanium carbide on Titanium carbide	ASTM F2423*	FE: ± 7.5° (Phase II) LB: ± 6.0° (Phase I) AR: ± 6.0° (Phase I) AL: 100 N (Phase I/II) F: 1.0 Hz	As published: Phase I then Phase II: 0.35 ± 0.3 mm^3^/MC*	Average size less than 0.2 µm

* Four wear tests were described in the SSED for the Prestige^®^ LP Cervical Disc; the maximum standard wear rate has been reported; ASTM = American Society for Testing and Materials; ISO = International Standards Organization; FE = flexion/extension; LB = lateral bending; AR = axial rotation; AL = axial load; F = frequency; SSED = Summary of Safety and Effectiveness Data; N/A = not available.

Few studies have reported impingement wear rates for cervical disc replacements and the data that do exist are primarily for polyethylene-on-cobalt-chrome designs. The summary of safety and effectiveness for LDR’s Mobi-C device [24] indicates the impingement wear rate for the cobalt chrome-on-cobalt chrome articulation was 0.23 ± 0.20 mg/MC and 0.87 ± 1.1 mg/MC for the superior and inferior endplates, respectively. Grupp et al. [28] reported an impingement wear rate of the active-L lumbar TDR and found the impingement wear rate to range from 0.06 ± 0.17 mm^3^/MC to 1.44 ± 0.54 mm^3^/MC, depending on the impingement conditions analyzed. For these designs, the idealized wear conditions lead to polyethylene wear rather than CoCr wear; therefore, comparing the wear rates between idealized and impingement for these designs is not possible.

Although third-body damage on retrieved TDRs has been reported [37], to our knowledge, there are no other reports of cervical disc bearings being tested under abrasive conditions. Third-body damage can be assessed using a variety of methods that have been suggested in the orthopaedic literature [18, 26, 33]. For this design, the combination of titanium plasma-sprayed endplates and the ceramic core made using titanium transfer relevant. However, for other total disc designs, this method of simulating third-body wear may not be appropriate.

For the idealized wear test, 65% of the particles produced were less than 1.0 µm. In comparison to published data for polymer-on-hard couples, four studies indicated that greater than 90% of the particles were less than 1.0 µm and reported average particle sizes from other studies ranged from 0.17 to 0.77 µm (Table 2). Only one publication provided a distribution for a PEEK-on-PEEK articulation, and it stated that 99% of the particles produced were less than 1.0 µm (Table 2). Overall, it appears the PEEK material, when articulated against ceramic or PEEK under cervical disc loading conditions, produces particles that are consistently bimodally distributed with the majority of particles occurring in the 1 to 2 µm and < 0.5 µm size ranges.

Overall, the wear performance of the PEEK-on-ceramic bearing under idealized and adverse in vitro wear testing conditions suggests the bearing couple may be robust for cervical total disc applications and promising for further clinical evaluation. The polymer-on-hard bearing performance appears microabrasive in nature and not substantially different from other traditional polymer-on-hard bearing couples. However, no in vitro assessment of wear can replicate all of the clinical conditions the end device will experience. Ultimately, the in vivo response to wear debris from the couple will need to be monitored to understand the clinical impact of the debris generated.
